# The Role of Specific Mitogen-Activated Protein Kinase Signaling Cascades in the Regulation of Steroidogenesis

**DOI:** 10.1155/2011/821615

**Published:** 2011-01-05

**Authors:** Pulak R. Manna, Douglas M. Stocco

**Affiliations:** Department of Cell Biology and Biochemistry, Texas Tech University Health Sciences Center, Lubbock, TX 79430, USA

## Abstract

Mitogen-activated protein kinases (MAPKs) comprise a family of serine/threonine kinases that are activated by a large variety of extracellular stimuli and play integral roles in controlling many cellular processes, from the cell surface to the nucleus. The MAPK family includes four distinct MAPK cascades, that is, extracellular signal-regulated kinase 1/2 (ERK1/2), p38 MAPK, c-Jun N-terminal kinase or stress-activated protein kinase, and ERK5. These MAPKs are essentially operated through three-tiered consecutive phosphorylation events catalyzed by a MAPK kinase kinase, a MAPK kinase, and a MAPK. MAPKs lie in protein kinase cascades. The MAPK signaling pathways have been demonstrated to be associated with events regulating the expression of the steroidogenic acute regulatory protein (StAR) and steroidogenesis in steroidogenic tissues. However, it has become clear that the regulation of MAPK-dependent StAR expression and steroid synthesis is a complex process and is context dependent. This paper summarizes the current level of understanding concerning the roles of the MAPK signaling cascades in the regulation of StAR expression and steroidogenesis in different steroidogenic cell models.

## 1. Introduction

For the control of diverse signaling in response to the extracellular milieu, cells develop sophisticated tools to transmit the appropriate signals and thereby orchestrate the responses. The signaling mechanism of an agent involves effector-receptor coupling, production of second messengers, activation of protein kinases, and distribution of these transducers to specific intracellular targets. Mitogen-activated protein kinases (MAPKs) are the serine/threonine kinase family of conserved enzymes that are considered to be the central building blocks in the intracellular signaling networks [1–5]. There are more than a dozen of MAPKs known in mammals, and these enzymes exist in several isoforms (Table 1). Mammals express four distinctly regulated groups of MAPKs, that is, extracellular signal-regulated kinase 1/2 (ERK1/2), p38, c-Jun N-terminal kinases/stress-activated protein kinases (JNK/SAPKs), and ERK5/Big MAPK (BMK1) [2, 3, 6]. Each of these four MAPK cascades consist of at least three tiers of protein kinases that are consecutively activated by phosphorylation events: MAPK kinase kinase (MAPKKK or MAP3K or MEKK) activates MAPK kinase (MAPKK (MKK or MEK) or MAP2K), which in turn activates MAPK. The latter phosphorylates a large array of substrates, including MAPK-activating protein kinases (MAPKAPKs) [7–9]. Even so, the different tiers are composed of many similar isoforms that can be activated by more than one MAPK, increasing the complexity and diversity of MAPK signaling. Substrates, regulation, and function of different MAPKs have been discussed in many review articles [1, 2, 4, 5, 9–11], and will not be elaborated upon in great detail here. MAPKs are important signal transducing enzymes that are involved in transmitting signals from a wide variety of extracellular stimuli including those of growth factors, hormones, cytokines, and neurotransmitters. In fact, MAPKs are major components of signaling pathways regulating a large array of intracellular events, such as proliferation, differentiation, development, acute signaling in response to hormones, stress response, programmed cell death, gene expression, and steroidogenesis [1, 2, 12–14]. Noteworthy, however, the role of the MAPK signaling pathways in steroidogenesis is poorly understood as a consequence of conflicting reports demonstrating stimulation, inhibition, or no effect in different steroidogenic cells [12, 15–18]. 

The steroidogenic acute regulatory protein (StAR) mediates the rate-limiting and regulated step in steroid hormone biosynthesis, that is, the transfer of the substrate for all steroid hormones, cholesterol, from the outer to the inner mitochondrial membrane [19–25]. As such, this protein plays a crucial role in the regulation of steroid hormones required for life itself, in the case of adrenal steroids, and for maintaining normal reproductive function, in the case of gonadal steroids. StAR is a rapidly synthesized, labile phosphoprotein, whose expression, activation and extinction are regulated by PKA, PKC, and a host of other signaling pathways (reviewed in [23, 25–27]). The StAR protein is localized to the mitochondria and consists of several forms of a newly synthesized 30-kDa protein, which has a 37 kDa precursor form containing an N-terminal mitochondrial targeting sequence [28, 29]. StAR is primarily associated with steroid producing tissues, and studies have demonstrated a tight correlation between the synthesis of StAR proteins and the synthesis of steroids. The compelling evidence for the critical role StAR in the regulation of steroidogenesis has been illustrated by the targeted disruption of the StAR gene and by the study of patients suffering from lipoid congenital adrenal hyperplasia, in which both adrenal and gonadal steroid biosyntheses are severely impaired due to mutations in the StAR gene [30–33]. In the adrenal and gonads, cAMP mediated mechanisms predominantly regulate expression of the StAR protein and steroid synthesis that involve transcriptional and translational induction. Conversely, transcriptional and/or translational inhibition of StAR expression results in a dramatic decrease in steroid biosynthesis, with the exception of approximately 10%–15% of steroid production that appears to occur through StAR-independent events [34–38]. It should be noted that phosphorylation of StAR is required to produce optimal cholesterol transferring ability of the StAR protein in steroid biosynthesis. Two putative PKA phosphorylation sites at serine 56/57 and serine194/195 have been identified, in murine and human StAR respectively, and mutations in these sites demonstrated the functional importance of the latter in the biological activity of StAR [39, 40]. While the cAMP/PKA pathway is unquestionably the major signaling pathway for trophic hormone-stimulated StAR expression and steroid biosynthesis, it has been well established that the MAPK signaling cascades play important roles in regulating these processes. The purpose of this paper is to summarize the findings of a number of laboratories, including our own, that have examined the roles of four distinct mammalian MAPK cascades in regulating steroidogenesis, and where evidence exists, on StAR expression, in steroidogenic cells.

## 2. The MAPK Signaling Cascade

The MAPK signaling cascade involves activations of several transmembrane signaling molecules, cytoplasmic protein kinases, and a network of interacting proteins, and these, in turn, regulate almost all cellular processes, from gene expression to cell death [1, 3, 7, 14, 41, 42]. Each of these cascades consists of several tiers of protein kinases that sequentially activate each other upon phosphorylation. Activation of each of the MAPK pathways is mediated by small GTP-binding proteins (e.g., Ras, Rap, or other oncoproteins), or by scaffold proteins or by adaptor molecules [41, 43, 44]. These events allow the transmission of extracellular signals to appropriate intracellular targets either directly or through three-tiered kinase modules [8, 10, 41]. MAPKs are activated by phosphorylation cascades that contain at least two upstream protein kinases. In all MAPK cascades, the kinase immediately upstreaming of the MAP kinase is a member of the MAP/ERK kinase (MEK or MKK) family. These are dual-specificity enzymes that are capable of phosphorylating serine/threonine and tyrosine residues in their MAP kinase substrates in order to activate the protein's activity [8, 45–47]. However, it is known that MAP kinases have overlapping substrate specificities. The MEKs are also activated by phosphorylation on serine or threonine residues in their activation loops. There are several and diverse MEK kinases (MEKKs) that activate MEKs [4, 9]. In contrast, the activity of MAPKs is negatively regulated by MAPK phosphatases, a group of dual-specificity phosphatases that remove phosphate from serine/threonine or tyrosine residues and thereby inactivate MAPKs for controlling signals [48–50]. 

**Figure 1:**
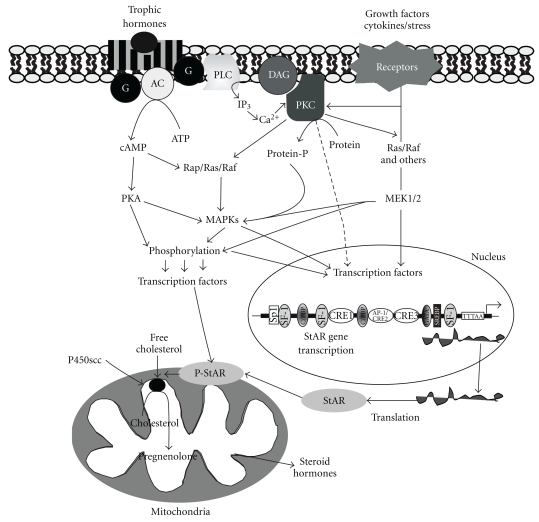
A schematic model illustrating the involvement of multiple signaling in MAPK-mediated regulation of StAR expression and steroidogenesis. Interaction of trophic hormones with their specific membrane receptors results in the activation of G proteins (G), which, in turn, stimulate adenylate cyclase (AC) that catalyzes the production of cAMP from ATP. cAMP then activates PKA and results in the phosphorylation of transcription factors involved in StAR gene transcription. The binding of growth factors results in activation of receptor tyrosine kinase and mediates biological functions via a number of mechanisms, including receptor autophosphorylation, receptor clustering, and phosphorylation of intracellular proteins. This leads to the activation of a cascade of protein kinases including Ras/Raf, and other related kinases. These protein kinases, in turn, activate different transcription factors, including CREB/ATF-1, cFos, and cJun. Phosphorylation of these transcription factors results in the transcriptional regulation of the StAR gene and, thus, steroid biosynthesis. The PKA and PKC signaling pathways can directly or indirectly activate transcription factors, and both of these pathways are involved in the MAPK mediated regulation of steroidogenesis. Furthermore, cAMP and/or different factors are also capable of activating a cascade of protein kinases (Rap/Ras/Raf or other related oncoproteins) leading to a number of MAPK signaling cascades, which have been demonstrated to play important roles in regulating StAR expression and steroid biosynthesis in steroidogenic tissues.

The MAPK signaling pathway has long been implicated in the regulation of cell cycle progression, is used as a biochemical marker in evaluating the mitogenic effects of a variety of stimuli, and is a point of convergence for diverse signaling pathways [12, 15, 16, 51, 52]. In mammals, four distinct MAPK cascades (ERK1/2, p38, JNK/SAPK, and ERK5/BMK1) are primarily activated by specific MAPKKs, that is, MEK1 and MEK2 for ERK1/2, MKK3/6 for p38, MKK4/7 for JNK/SAPK, and MEK5 for ERK5/BMK1 [1, 2, 41]. Nevertheless, each MAPKK can be induced by many MAPKKKs, and presumably each MAPKKK confers responsiveness to discrete signals [9, 53]. The differential expression, activation, and substrate specificity of these MAPKs suggest their varying physiological functions in different cellular contexts. A substantial body of evidence indicates that MAPK signaling cascades are activated by a large number of extracellular signals and play pivotal roles in regulating the steroidogenic response. As such, a variety of exogenous stimuli (e.g., hormones, growth factors, and cytokines) that influence different MAPK cascades exhibit diverse effects on StAR expression and steroidogenesis in different steroidogenic cell models (Figure 1; [12, 15–17, 54, 55]).

### 2.1. ERK1/2 Signaling and Its Role in StAR Expression and Steroidogenesis

The ERK1/2 signaling cascade is the most widely studied member of the MAPKs. ERK1 (p44 MAPK) and ERK2 (p42 MAPK) are highly conserved throughout eukaryotic cells and bring together transmembrane receptors and a network of various proteins and integrating signals to control many cellular processes, including cell survival, differentiation, tumor progression, and invasion [4, 7, 56–58]. There are also alternatively spliced forms of ERKs, namely, ERK1b (46 kDa), ERK1c (42 kDa), and ERK2b (41 kDa). ERKs are ubiquitously expressed to varying extents in different tissues. Both ERK1 and ERK2 are activated by numerous extracellular stimuli, including growth factors, cytokines, transforming agents, and ligands that act via G protein-coupled receptors (GPCRs) and tyrosine kinase receptors. The two phosphoacceptor sites, tyrosine and threonine, in the activation loop (TEY) are phosphorylated in order to activate the ERK1/2 signaling cascade, which occurs exclusively through MEK1 and MEK2. ERKs also phosphorylate several substrates (e.g., ribosomal S6 kinase (RSK), the MAPK/SAPK-activated kinase (MSK)) either in the cytosol (e.g., PLA2) or in the nucleus (e.g., Elk1) [1, 4, 9, 11]. However, this activation requires adaptor proteins that are linked to the guanine exchange factors (GEFs) of GTP-binding proteins. Following stimulation, recruitment of the adaptor protein-GEF complex to the plasma membrane results in the induction of Ras or related molecules, which then transmit signals to the MAP3K level of the cascade (Raf-1, B-Raf, A-Raf, TLP-2, and MEKK1) [1, 9, 59]. This, however, is an oversimplification of the ERK1/2 signaling module. Generally, the three-tiered system of the ERK1/2 signaling cascade is rather complex and involves many MEKKs that are capable of phosphorylating a number of MEKs and thereby activating several MAP kinases and have been discussed elsewhere [1, 2, 4, 11, 41].

The involvement of the ERK1/2 pathway in steroidogenesis has been widely studied. This signaling pathway is involved in regulating StAR expression and steroid biosynthesis, but several seeming contradictions have been reported in different steroidogenic tissues [12, 15–17, 54, 60–62]. For example, it has been demonstrated that activation of ERK1/2 by hCG increases StAR expression and testosterone production while inhibition of ERK1/2 by U0126 results in decreases in these parameters in hCG-stimulated primary cultures of immature rat Leydig cells [61]. In contrast, ERK1/2 inhibition enhances StAR expression as induced by LH, insulin-like growth factor-1 (IGF-1), transforming growth factor-*α*, and interleukin-1*α* (IL-1*α*) but decreases steroid levels [2, 12, 52, 62, 63]. It has been reported that steroid biosynthesis induced by hCG and fibroblast growth factor-9 (FGF-9) is mediated through the involvement of Ras-MAPK and PKA signaling in mouse Leydig cells [64, 65]. We have demonstrated that the ERK1/2 signaling cascade plays key roles in PKA-mediated (activated by (Bu)_2_cAMP and hCG)- and PKC-mediated (activated by PMA) regulation of steroidogenesis [12, 52, 66]. All these agents activate ERK1/2; however, they have diverse effects on StAR expression and steroid synthesis in conjunction with ERK1/2 inhibition by either PD98059 or U0126. In particular, whereas the inhibition of ERK1/2 increases cAMP/hCG-stimulated StAR expression, it decreases PMA-mediated StAR levels. Nonetheless, progesterone levels were diminished in all cases. The decrease in steroid synthesis was not associated with attenuation of the cytochrome P450 side chain cleavage (P450scc) and 3*β*-hydroxysteroid dehydrogenase (3*β*-HSD) enzyme activities, as ERK1/2 inhibition had no effect on 22R-hydroxycholesterol-mediated steroid synthesis [12]. Noteworthy, the increases in PMA, (Bu)_2_cAMP, and hCG-induced StAR expression and progesterone levels were inversely correlated with the levels of a negative transcription factor, namely, dosage-sensitive sex reversal, adrenal hypoplasia congenita, critical region on the X-chromosome, gene 1 (DAX-1) [12, 52, 66, 67]. While U0126 attenuates DAX-1 expression, this inhibition can be reversed by PMA but not by (Bu)_2_cAMP/hCG [12, 66]. In fact, the differential effects of U0126 on PKA- and PKC-mediated steroidogenesis is mediated, at least in part, by alterations in DAX-1 expression in mouse Leydig cells. Furthermore, studies have shown that both (Bu)_2_cAMP and PMA can elevate the levels of scavenger receptor class B type 1 (SR-B1, a high-density lipoprotein (HDL) receptor that binds various lipoprotein particles and facilitates cellular cholesterol influx) and that ERK1/2 inhibition decreases SR-B1 expression [12]. The binding of HDL to SR-B1 has been reported to stimulate the ERK1/2 pathway following Ras activation [68]. SR-BI is involved in importing cholesterol into cells that will eventually be provided to the mitochondria for steroid biosynthesis [52, 69]. Thus it is plausible that the decrease in steroid synthesis caused by U0126 (during conditions of elevated StAR) is due to a reduction in cholesterol availability to the mitochondria. Recent studies have shown that activation of ERK1/2 at the mitochondria plays an important role in Leydig cell steroidogenesis [70, 71]. Specifically, ERK1/2 is capable of phosphorylating StAR at serine 232 in the presence of cholesterol, demonstrating that StAR is a substrate for ERK1/2 and that a mitochondrial kinase complex is essential for maximum cholesterol transferring capacity of the StAR protein in steroid synthesis.

In granulosa cell-derived rLHR-4 and rFSHR-17 cell lines, treatments with LH/hCG and FSH, respectively, increase ERK1/2 phosphorylation, StAR expression, and progesterone synthesis [16, 17, 51, 72]. Conversely, gonadotropin-stimulated StAR and steroid levels have been shown to be further augmented following inhibition of ERK1/2 both by PD98059 and U0126, suggesting that the induction of LH/hCG- and FSH-induced steroidogenesis involves downregulation of the steroidogenic machinery including the ERK1/2 cascade. Studies have also shown that while PD98059 and U0126 have no effects on LH/hCG, insulin, or IGF-1-induced steroidogenesis, they decrease (Bu)_2_cAMP, cholera toxin and forskolin stimulated progesterone production [54, 60]. Further, it has been reported that FGF-8 mediated ERK1/2 activity is associated with an attenuation of estradiol production in rat granulosa cells [73]. Also, ERK1/2 activation results in differential effects on LH-induced StAR and Cyp17 expression in bovine theca cells. Activation results in an increase in StAR and a decrease in Cyp17 that in turn results in an elevation in progesterone and a reduction in androsterone levels, processes in which DAX-1 and steroidogenic factor 1 (SF-1) play pivotal roles [17]. Likewise, administration of hCG *in vivo* or treatment with gonadotropins/cAMP *in vitro* is associated with down-regulation of Cyp19a1 and upregulation of StAR and Cyp11a1 mRNA expression in both mural and cumulus granulosa cells of mouse preovulatory follicles [74]. These events have been shown to be tightly regulated with steroid biosynthesis and involve ERK1/2-dependent signaling. Recently, the activation of ERK1/2, as well as other MAPK signaling, by leptin, has been linked to decreases in basal and cAMP/PKA stimulated StAR expression and progesterone synthesis without affecting the levels of the P450scc and 3*β*-HSD enzymes [75]. Additionally, treatment with metformin (a drug that is widely used for treating infertility in women with polycystic ovary syndrome (PCOS)) causes an activation of ERK1/2 that results in suppression of insulin-stimulated P450 aromatase mRNA expression and activity [76]. In theca cells isolated from PCOS patients, increases in Cyp17 expression and androgen biosynthesis are connected to an attenuation of ERK1/2/MEK1/2 activity by PD98059, suggesting the involvement of MAPK signaling in the pathogenesis of this disease [77]. Prostaglandin F_2*α*_ (PGF_2*α*_), an agent that influences luteal regression through the induction of the phospholipase C/diacylglycerol/PKC pathway, induces phosphorylation of ERK1/2, which, in contrast, decreases basal and LH/hCG-stimulated StAR expression and progesterone synthesis in human granulosa-luteal cells [78]. These effects of PGF_2*α*_ in the steroidogenic response can be reversed following inhibition of MEK activity by PD98059. Moreover, the inhibitory effect of PGF_2*α*_ on StAR expression and steroidogenesis, in rat luteal cells, is modulated by the negatively acting transcription factors, DAX-1 and ying yang 1 [18, 79]. These results demonstrate the multiple effects of the ERK1/2 signaling cascade in StAR expression and steroidogenesis in gonadal cells.

In mouse Y-1 adrenocortical tumor cells the induction of forskolin-mediated corticosterone synthesis is dependent upon the activation of ERK1/2 and is correlated with phosphorylation of SF-1 and increased StAR gene transcription [15]. Accordingly, inhibition of ERK1/2, by either PD98059 or U0126, decreases StAR expression and steroid production without affecting P450scc enzyme activity. Increasing evidence demonstrates that ACTH and angiotensin II (Ang II) can elevate phosphorylation of p44/p42 MAPKs and result in increases in StAR expression and steroid synthesis that involve PKC signaling and Ras/Raf-1 kinase [80–83]. The induction of orexins A and B (neuropeptide hormones) mediated StAR expression and steroid synthesis is mediated by a number of MAPK cascades including EKR1/2 activation, where the latter requires multiple G-protein signaling pathways in human H295R adrenocortical cells [55, 84]. It has been demonstrated that while proopiomelanocortin (POMC) fragments, 1-28-POMC and 1-48-POMC, modulate cellular proliferation, they decrease adrenal steroidogenesis by activating ERK1/2 signaling [85]. Chronic administration of ACTH induces the phosphorylation of ERK1/2 that occurs in parallel with adrenal corticosterone synthesis in adult rats [82]. In both H295R and primary cultures of human adrenocortical cells, treatment with adipokines up-regulates the ERK1/2 cascade and results in increases in StAR expression and aldosterone synthesis, processes that do not require cAMP/PKA signaling [86, 87]. Taken together, it seems clear that the ERK1/2 signaling cascade plays diverse roles in regulating StAR expression and steroidogenesis. These roles could be a result of the existence of multiple signal transduction pathways that display differences in receptor-effector coupling between tissues and species.

### 2.2. p38 MAPK Signaling and Its Role in Steroidogenesis

The p38 MAPK signaling cascade is thought to participate in the response of cells to stress. Four members of the p38 MAPK family have been cloned and named p38*α* (MAPK14), p38*β* (p38-2), p38*γ* (ERK6 or SAPK3), and p38*δ* (SAPK4) and share approximately 60% homology in their amino acid sequences [10, 88–91]. Also, several alternatively spliced isoforms of p38 MAPK include Mxi2 (identical to p38*α*) and Exip. P38 MAPKs contain a Thr-Gly-Tyr activation loop sequence (TGY). They are activated by dual phosphorylations on threonine and tyrosine residues in response to numerous stimuli, including cytokines, hormones, GPCRs, heat shock, and other stresses and play important roles in controlling many cellular functions [42, 53, 92, 93]. P38*α* is expressed in most cells; however, expression of other isoforms is tissue specific. Cellular distribution, activation, and substrate specificity of p38 MAPKs result in diverse biological functions. Once activated, p38 MAPKs either transmit the signals via a three-tier cascade or phosphorylate other regulatory molecules such as PLA2, heat shock proteins, and transcriptions factors (c-Jun, ATF-2, CREB, CHOP, NF-kB, and others) [8, 42, 92, 94]. Substrates of p38 MAPK include MAPK-activated protein kinases (MKs), that is, MK2, MK3, and MK5 (reviewed in [1, 53, 95, 96]). There are also several distinct kinases at the MAP3K level of the p38 MAPK cascade, including MLK2, MLK3, TPL2, dual leucine zipper-bearing kinase, ASK1, MAP three kinase 1, and TAK1 [91, 97, 98]. Studies have reported the existence of p38 signaling crosstalk with other MAPK cascades. For instance, the p38 MAPK pathway causes rapid inactivation of the ERK1/2 cascade mediated by PP2A [99]. The p38 MAPK pathway is involved in tissue homeostasis and several pathologies ranging from inflammation and the immune response to cancer and neurodegenerative diseases [93, 100, 101]. 

The p38 MAPK signaling cascade has been implicated in regulating steroidogenesis. It has been demonstrated that IL-1*α* activates p38 MAPK and that this event is associated with StAR expression and testosterone synthesis in immature rat Leydig cells [102, 103]. The involvement of p38 MAPK in steroidogenesis is further assessed by observations in which inhibition of its activity, either by SB203580 or PD169316, results in the coordinate suppression of StAR and steroid levels. IL-1*α* is also capable of phosphorylating CREB and Fos/Jun through the activation of a p38 substrate which is a RSK family member (RSK-B kinase, also called MSK2), suggesting that this process may play a role in the differentiation of immature into adult Leydig cells [102]. In addition, it is worth noting that IL-1*α* also activates ERK1/2 and inhibition of the latter by U0126 augments expression and phosphorylation of the StAR protein but decreases androgen synthesis by dissipating the mitochondrial electrochemical potential [63]. These findings suggest that a number of MAPK signaling events differentially influence IL-1*α*-mediated steroidogenesis in mouse Leydig cells. 

Gonadotropins have been shown to activate both p38 and ERK1/2 MAPKs and result in varying effects on StAR expression and steroidogenesis in ovarian granulosa cells [16, 17, 51, 104]. Studies have demonstrated that interference of p38 (by SB203580) and ERK1/2 (by PD98059 and U0126) activity is associated with increases in LH/hCG/FSH mediated StAR expression and progesterone synthesis [16, 51, 104]. In addition, inhibition of p38 decreases both P450arom and estradiol synthesis, and these events are tightly correlated with liver receptor homolog-1 and DAX-1 expression [104], demonstrating that p38 targets these transcription factors in regulating steroidogenesis. Heat shock protein HSP-27 is identified as a downstream phosphorylation target of FSH- and forskolin-mediated p38 MAPK activation [105]. In granulosa cell-oocyte cocultures, both bone morphogenetic protein-2 (BMP-2) and BMP-4 exert differential effects on FSH mediated regulation of steroidogenesis through the activation of p38 MAPK [106]. Indeed, both BMP-2 and -4 increase FSH-mediated P450arom mRNA expression and estradiol production but decrease StAR and progesterone levels. 

In primary cultures of rat adrenal glomerulosa cells, Ang II activates the p38 and ERK1/2 signaling pathways and results in increases in StAR expression, steroidogenic enzymes, and steroid synthesis [83, 107]. Concurrently, Ang II inhibits protein synthesis by enhancing p27^kip1^ expression (a protein known to block the cell cycle in the G1 phase). The effects of Ang II can be reversed through inhibition of p38 activity by SB203580, suggesting that Ang II plays an important role in adrenal physiology. Accumulating evidence indicates that activation of the p38 MAPK signaling cascade is linked to the aging-induced, oxidative stress-mediated suppression of steroidogenesis in adrenal cells [108–110]. Alternatively, inhibitors of p38 MAPKs (by either SB203580 or SB202190) and antioxidants (reactive oxygen species (ROS) scavengers MnTMPyP and N-acetyl cysteine) have been shown to restore corticosterone synthesis in cells from aged rats. These findings indicate that the stress-mediated inhibition of steroid biosynthesis involves the activation of the p38 MAPK pathway in the adrenals during the course of aging [110]. Also, intense phospho-p38 MAPK immunoreactivity has been detected in human brains of postmortem patients afflicted with Alzheimer's disease [111, 112], indicating that p38 could be involved in the pathogenesis of this disease.

### 2.3. JNK/SAPK Signaling and Its Role in Steroidogenesis

JNK/SAPKs are also considered as stress-activated MAPKs; however, they are different from p38 MAPKs [1, 113, 114]. Isolation and subsequent characterization of cDNAs encoding these enzymes revealed three genes encoding proteins with 10 or more alternatively spliced forms. Three main isoforms, that is, JNK1/SAPK*γ* (46 kDa), JNK2/SAPK*α* (54 kDa), and JNK3/SAPK*β* (52 kDa), are approximately 85% identical in their core catalytic domains [115, 116]. Whereas JNK1/2 MAPKs are ubiquitously expressed, JNK3 is primarily localized to neuronal tissues, testis and cardiac myocytes. JNK/SAPKs are activated by cytokines, UV irradiation, growth-factor deprivation, agents that interfere with DNA and protein synthesis, as well as other stressors. Similar to other MAP kinases, the activity of JNK/SAPKs is dependent upon phosphorylation on tyrosine and threonine residues, which are separated by a proline to generate the TPY motif in the activation loop. The resulting signals are then transmitted to three-tier cascades either directly or through MAP4Ks [117, 118]. Several kinases are phosphorylated in these cascades [1, 41]. However, two MEK family members, MKK4 (SEK1, MEK4, JNKK1, and SKK1) and MKK7 (MEK7, JNKK2, and SKK4), are predominantly involved in the JNK/SAPK cascade for signal integration [117–119].

Several lines of evidence demonstrate the involvement of the JNK/SAPK signaling cascade in steroidogenesis. For example, tumor necrosis factor-*α* (TNF*α*) activates JNK/SAPK; however, the latter is associated with decreases in basal and cAMP-induced steroidogenesis by reducing Cyp17 gene expression in MA-10 cells [120]. TNF*α* also decreases the phosphorylation of ERK1/2 while simultaneously increasing the abundance of cJun as well as increasing AP-1 binding activity, suggesting the involvement of a number of MAPKs in TNF*α* signaling. Therefore, the activation of JNK/SAPK and ERK1/2 MAPKs appears to play a mutually antagonistic role in TNF*α*-mediated steroidogenesis. In rat R2C Leydig tumor cells, bisphenol A (BPA), an endocrine disruptor, is capable of activating JNK/SAPK and results in an elevation in aromatase activity and an attenuation of testosterone synthesis by targeting both CREB and Akt [121]. Increasing evidence demonstrates that steroidogenesis decreases in aging, a time when the levels of reactive oxygen species (ROS) increase. As a consequence, a number of ROS, such as superoxide anion (O_2_^−^) and hydrogen peroxide (H_2_O_2_), have been involved in the repression of testicular StAR expression and steroid synthesis [122–124]. In K28 mouse Leydig cells, the inhibition of ROS-mediated StAR, P450c17 mRNA, and steroid levels is mediated, at least in part, through the activation of JNK/SAPK MAPKs and subsequent upregulation of c-Jun [124]. These events cause a repression in the trans-activation potential of Nur77 on steroidogenic enzyme genes and result in decreases in StAR expression and steroidogenesis. 

In human granulosa cells, leptin activates a number of MAPKs including the JNK/SAPK signaling pathway where it decreases cAMP-induced StAR protein expression and progesterone synthesis [75]. Likewise, BMPs can induce phosphorylation of several MAPK cascades, exhibit varying actions on steroidogenesis, and play important roles in ovarian follicular growth and maturation [73, 125, 126]. Also, an oocyte derived factor, FGF-8, has been shown to interact with BMPs, activate the JNK/SAPK and ERK1/2 signaling cascades and subsequently regulate FSH-induced steroidogenesis in a rat granulosa cell-oocyte coculture system [73]. Altogether, the JNK/SAPK signaling cascade is fundamentally connected with stress-related responses and the activation of JNK/SAPK decreases the steroidogenic response in a number of steroidogenic tissues.

### 2.4. ERK5/BMK1 Signaling and Its Role in Steroidogenesis

The ERK5/BMK1 is the largest known MAP kinase (~110 kDa) family member. The signaling pathway leading to ERK5 activation is poorly understood in comparison with other MAPKs as a consequence of conflicting findings in the literature [127–130]. Importantly, the C-terminus of ERK5 contains 10 consensus MAP kinase phosphorylation sites which can be autophosphorylated [131]. ERK5 is ubiquitously expressed and its activity is regulated by a variety of proliferative (growth factors, phorbol ester, serum, and lysophosphatidic acid) and cell-stressing (H_2_O_2_, sorbitol, and UV irradiation) agents [132, 133]. The mechanism of upstream activation of the ERK5 cascade has not been fully defined. This mechanism may include the action of adaptor proteins (e.g., LAD), protein tyrosine kinases, and WNK1 [134–136]. These components have been shown to induce a number of kinases at the level of MAP3K, including MEKK2/3, TPL2, and MLTK [135, 137–139]. These kinases then phosphorylate MEK5 (an upstream component of ERK5) on serine and threonine residues [127]. MEK5 then activates ERK5 on threonine and tyrosine residues in the loop sequence motif (TEY) identical to ERK1 and ERK2. However, ERK5 cannot be phosphorylated by MEK1/2, and MEK5 does not phosphorylate ERK1/2. Another substrate of ERK5 is the serum and glucocorticoid-inducible kinase, which may serve as a MAPKAPK of this cascade, allowing for the possible involvement of a five-tier cascade [140]. ERK5 can affect a number of cellular activities (e.g., cellular proliferation, differentiation, and motility) by phosphorylating many transcription factors, including MADs box, c-Myc, c-Fos, myocyte enhancer factors 2A and C, and SAP1a [133, 141–143]. 

Relatively little is known regarding the role of ERK5 in steroidogenesis. Recently, it has been reported that orexins A and B activate ERK5 and concomitantly increase expression of the StAR protein and steroidogenic enzyme genes in H295R cells [55, 84]. These agents can also augment cortisol secretion in primary cultures of adrenal cells. The effects of orexins on ERK5 phosphorylation have been demonstrated to be similar to those of Ang II and are mediated by multiple G-protein signaling pathways. However, orexins simultaneously activate the ERK1/2 and p38 MAPK signaling cascades [55], indicating that the regulation of orexin-mediated StAR expression and steroidogenesis involves several MAPKs in adrenal cells. Information that the activation of the ERK5 signaling pathway in gonadal cells is linked to steroidogenesis is currently lacking.

## 3. Other MAP Kinases

ERK3, ERK4, ERK7, NLK, MOK, and others are considered as atypical MAPKs (Table 1). Regulation, structure, and substrate specificity of these MAPKs have been described in a recent review [5]. The roles of these atypical MAPKs in steroidogenesis remain to be elucidated.

## 4. Conclusions

The MAPK signaling cascades are the major components of pathways controlling a wide variety of cellular processes, including embryogenesis, gene expression, acute responses to hormones, cell survival, and apoptosis and are of critical importance in the transduction of extracellular signals in cells. Noteworthy, the MAPK cascade has been implicated in the pathogenesis of a number of human disorders, including Alzheimer's disease, Parkinson's disease, and many cancers. Indeed, the multiple effects of MAPKs could be a result of the involvement of a wide variety of substrates that include protein kinases and phosphatases, transcription factors, cytoskeletal elements, and other signal-regulated molecules. The studies summarized here have emphasized the roles of ERK1/2, p38 MAPK, JNK/SAPK, and ERK5 MAPKs in the regulation of StAR expression and steroidogenesis in different steroidogenic tissues. The StAR protein plays an indispensable role in the production of steroid hormones required for bodily homeostasis and normal reproductive development and function. Based on the results available at this time, it is obvious that the MAPK signaling cascades play diverse roles in controlling StAR expression and steroid biosynthesis in tissue-, stimulus-, and pathway-specific manners. These processes appear to be dependent on receptor-effector coupling, signaling crosstalk, and/or other factor(s) involved in steroidogenesis. Moreover, under specific circumstances, the regulation of MAPK-dependent StAR expression and steroidogenesis involves more than one MAPK signaling, and as a consequence different, and even opposing, effects of MAPKs can be seen in different steroidogenic cells. Additionally, different cells express distinct sets of transcription factors, and this diversity may account for the cell-type-dependent specificity of MAPK action. Given the physiological and pathological roles of the MAPK signaling pathways, elucidation of tissue- and disease-specific effects of each of the MAPK signaling cascades together with their downstream effectors requires better understanding. An abundance of molecular tools including high throughput genomic and proteomic technologies will undoubtedly provide valuable insights into these regulatory mechanisms.

## Figures and Tables

**Table 1:** Mammalian MAP kinases.

MAP kinase	Other names	Relevant information	Phosphorylation site motif
ERK1	P44 MAPK	~86% sequence similarity to ERK2, ubiquitously expressed, UO126 sensitive	TEY
ERK2	P42 MAPK	Ubiquitously expressed, UO126 sensitive	TEY
ERK3	P63, ERK*α*	Yields a single protein of 100 kDa possessing a C-terminal extension	SEG
ERK1b	ERK4	A separate gene	TEY
JNK1	SAPK*γ*	Several splice variants	TPY
JNK2	SAPK*α*	Several splice variants	TYP
JNK3	SAPK*β*	Several splice variants	TYP
P38*α*	P38, CSBP, SAPK2, MAPK14	Sensitive to SB203580	TGY
P38*β*	P38-2, MAPK11	Sensitive to SB203580	TGY
P38*γ*	ERK6, SAPK3, MAPK12	SB203580 insensitive	TGY
P38*δ*	SAPK4 or MAPK13	SB203580 insensitive	TGY
Mxi2		A splice form of P38*α*	TGY
Exip		A splice form of P38*α*	TGY
ERK5	BMK1	Cell proliferation, differentiation	TEY
ERK7	MAPK15	Cell proliferation	TEY
NLK	Nemo-like kinase	Ortholog of *C. elegans* LIT-1, relative of Drosophila nemo	TQE
MAK	Male germ cell- associated kinase	Expressed in testicular cells undergoing meiosis	TDY
MRK	MAK-related kinase	Ubiquitous in adult tissues	TDY
MOK		Phorbol ester sensitive	TEY
KKIALRE		Cdc2-related kinase	TDY
KKIAMRE		T, Y mutants activated in cells	TDY
